# Brain Potentials of Conflict and Error-Likelihood Following Errorful and Errorless Learning in Obsessive-Compulsive Disorder

**DOI:** 10.1371/journal.pone.0006553

**Published:** 2009-08-12

**Authors:** Anke Hammer, Andreas Kordon, Marcus Heldmann, Bartosz Zurowski, Thomas F. Münte

**Affiliations:** 1 Department of Neuropsychology, Otto-von-Guericke University of Magdeburg, Magdeburg, Germany; 2 Department of Psychiatry and Psychotherapy, University of Luebeck, Luebeck, Germany; 3 Department of Neurology, Otto-von-Guericke University of Magdeburg, Magdeburg, Germany; 4 Center for Behavioral Brain Sciences, Magdeburg, Germany; 5 Neuroimage Nord, University of Hamburg, Hamburg, Germany; University of Granada, Spain

## Abstract

**Background:**

The anterior cingulate cortex (ACC) is thought to be overacting in patients with Obsessive Compulsive Disorder (OCD) reflecting an enhanced action monitoring system. However, influences of conflict and error-likelihood have not been explored. Here, the error-related negativity (ERN) originating in ACC served as a measure of conflict and error-likelihood during memory recognition following different learning modes. Errorless learning prevents the generation of false memory candidates and has been shown to be superior to trial-and-error-learning. The latter, errorful learning, introduces false memory candidates which interfere with correct information in later recognition leading to enhanced conflict processing.

**Methodology/Principal Findings:**

Sixteen OCD patients according to DSM-IV criteria and 16 closely matched healthy controls participated voluntarily in the event-related potential study. Both, OCD- and control group showed enhanced memory performance following errorless compared to errorful learning. Nevertheless, response-locked data showed clear modulations of the ERN amplitude. OCD patients compared to controls showed an increased error-likelihood effect after errorless learning. However, with increased conflict after errorful learning, OCD patients showed a reduced error-likelihood effect in contrast to controls who showed an increase.

**Conclusion/Significance:**

The increase of the errorlikelihood effect for OCD patients within low conflict situations (recognition after errorless learning) might be conceptualized as a hyperactive monitoring system. However, within high conflict situations (recognition after EF-learning) the opposite effect was observed: whereas the control group showed an increased error-likelihood effect, the OCD group showed a reduction of the error-likelihood effect based on altered ACC learning rates in response to errors. These findings support theoretical frameworks explaining differences in ACC activity on the basis of conflict and perceived error-likelihood as influenced by individual error learning rate.

## Introduction

In addition to other characteristic symptoms of obsessive compulsive disorder (OCD), such as chronic doubt, repetitive controlling, ruminations, and reduced behavioral flexibility [1], a number of neuropsychological studies have revealed altered memory functions [2]–[7] and executive dysfunction [8]–[14]. Following a systematic review [15], memory deficits are the most consistently reported neuropsychological features of OCD patients. A strategic memory deficit has been described for non-verbal material [7] and verbal material [16]–[19]. Other studies suggested that OCD patients have reduced confidence in the correctness of their memory contents, which consequently affects memory-based decisions [20]–[26]. These findings indicate that memory problems in OCD might be related to executive aspects of memory, such as meta-memory decisions about whether or not an associatively retrieved item has indeed been encountered before, rather than to deficits within the memory system proper. For example, OCD-patients might not have a problem in memorizing their shopping list per se but due to controlling or repetitive thinking about the memorized items the actual memory performance is impaired which might lead to incorrectly added and forgotten items.

Here, we tested this hypothesis by comparing two different encoding strategies between OCD patients and control participants. We used brain potentials to further delineate the characteristics of cognitive control during memory processes in OCD by means of errorless (henceforth EL) and errorful (henceforth EF) learning [27]–[30]. During EF-learning, interfering and thus possibly conflicting items are presented in addition to the relevant stimulus. In contrast, in EL-learning only the target stimulus is presented and only this stimulus without any further conflicting stimuli is thus available for storage resulting in an improved memory performance for EL learning as compared to EF learning. Baddeley and Wilson [27] assumed that the worse memory performance after EF-learning is due to the increased activation level of false candidates which leads to interference in recall. This interference is thought to be absent (or greatly diminished) in the EL modus as only one stimulus had been presented during learning. In EF-learning, memory impaired patients may not be able to use the remaining implicit memory resources, because they are not able to differentiate between errors made during learning and the correct information [27]. Consequently, these patients benefit from EL-learning compared to EF-learning as errors are avoided during the studying phase.

Rodriguez-Fornells and colleagues [29] and Heldmann et al. [31] investigated EL and EF-learning in conjunction with the recording of brain potentials. In both studies a word stem completion task was used. In EF learning, the initial three letters of a word (e.g. C-O-M) were presented and the participants were asked to guess which word the experimenter had in mind. After some guesses (e.g. compare, computer, commission, comedy) the experimenter indicated the correct word (e.g. comedy). This procedure introduces errors during learning as described above. In contrast, during EL learning, the intended word is given right after the initial letters and errors are prevented during learning. Brain potentials were acquired during recognition of words and the participants had to indicate via button response whether a word was learned before or not (correctly identifying target words or correctly rejecting non-target words). Both studies demonstrated EL/EF effects in particular for response-locked brain potentials, which are thought to reflect aspects related to the memory decision. In particular, a short latency midfrontal phasic negativity peaking at about 50 ms after the memory decision (here button press) was found to be modulated by learning mode. This negativity showed the topographic and latency characteristics of the error-related negativity (ERN) [32]–[35] previously described in research on action monitoring. It's neural source was consistently found in the posterior medial frontal cortex, most likely the anterior cingulate cortex (ACC) as shown by brain potentials source localization studies [36]–[39] and error-related fMRI activity [40]–[42]; regions that are known to be involved in higher executive functions. Rodriguez-Fornells et al. [29] found the highest ERN amplitude for false alarms after EL-learning and intermediate-sized amplitudes for hits and false alarms in the EF condition. The smallest amplitude was found for hits in the EL condition and the ERN was absent for correct rejections. The modulation of the ERN amplitude in relation to memory decisions was interpreted as reflecting the activity of an internal monitoring device assessing the activation of the two possible decisions [29]. This interpretation places the occurrence of an ERN for memory decisions in the context of the conflict monitoring theory of the ERN [43] assuming that the activation depends on the product of current activations of concurrently available responses (here correct and guessed words). EF and EL learning was presented in an intermixed procedure which might lead to a rudimentary activation of non-targets in EL learning as well. In a further study in healthy participants, Heldmann et al. [31] presented EF and EL learning in blocked sessions and included additional new words during recognition which did not occur during learning. Thus there were more words that needed to be rejected (non-targets require a NO response) as compared to words to be recognized (targets require a YES response) resulting in an unequal ratio of NO and YES responses. In other words, the risk to make an error is increased for YES as compared to NO responses. Irrespective of the correctness of the response, Heldmann et al. [31] observed an ERN for items classified as learned before (i.e. YES-responses: hits and false alarms) as compared to items classified as not learned before (i.e. NO-responses: correct rejections and misses). These results lead to the argument that variations of the ERN amplitude in EL/EF-learning might be partially explained by the subjects' perceived likelihood of making an error. This interpretation was based on the error-likelihood model [44], [45], [but see also 46], which postulates that the activation of the ACC (and thus its electrophysiological counterpart, the ERN) is not modulated by the presence of conflict or the detection of an error per se but the perceived probability of making an error (here the ratio of YES/NO responses).

There are alternative theoretical approaches explaining modulations of the ERN: the error detection approach [33] and its extension, the reinforcement learning model [47]. Following the reinforcement model an error is understood as a negative reinforcement signal processed within the mesencephalic dopaminergic system. The resulting changes in dopaminergic activity are used for further adaptation of behavior in order to avoid errors in the future. For the given investigation we would like to focus on the error likelihood model for two reasons. First, this model was a good candidate explaining the findings of Heldmann et al. [31] and second, an extension of the model takes individual differences into account. This extension of the error-likelihood model [48], [49] showed that the model can account for individual differences related to error-likelihood, prediction of error consequences, and conflict effects in ACC. Individuals with a high learning rate within ACC (i.e. learning from errors, adapting the following behavior to circumvent unwanted consequences in future) resemble the known patterns of the model: the higher the probability to commit an error the higher the activity within ACC. In contrast, individuals with a slow learning rate and thus a reduced ability within ACC to learn from errors showed smaller error-likelihood effects whereas conflict effects increased. This finding indicated an inverse relationship between conflict and error-likelihood effects dependent on the error learning rate [48], [49]. Such a result is of specific interest for OCD patients as previous results suggested an impaired cognitive control and thus possibly altered learning rates in ACC following errors. Previous studies in OCD patients found increased amplitudes of the ERN [50]–[56], [see 57 for partly inconsistent results], which has been interpreted as evidence for an increased action monitoring compared to controls. This interpretation has been corroborated by neuroimaging data showing hyperactivity of the ACC in OCD patients which was positively correlated with symptom severity [58], [59].

None of these studies modulated the perceived error-likelihood via different learning modes. We used brain potentials to assess executive aspects of memory in OCD by contrasting EF and EL-learning. OCD-patients are continuously monitoring their behaviour but still remain with the feeling of erroneous actions and states [60]. The present paradigm is of specific interest because (a) we can compare errors in conflicting (EF) with conflict-reduced circumstances (EL) and additionally (b) correct responses in differential conflicting situations. These differences in conflict processing can be evaluated based on different learning modes, i.e. recognition following errorful learning as compared to errorless learning is thought to be conflicting based on the additional interfering material. Here, influences of error-likelihood can be evaluated on the basis of the response options (Yes vs. No response) depending on the ratio of target and non-target items (see also [31]).

In healthy subjects, we expected to replicate the basic findings for EL and EF-learning [29], [31]. However, in OCD patients we expected an increased ERN for EL-learning compared to a control group [50]–[55], [57]. EF-learning increases the interference in later recognition – and thus the action monitoring system is challenged in particular. The present study was designed to answer the question which one of the following is true for OCD patients: Either the dysfunctional action monitoring system in OCD patients (1) is overactive resulting in increased ERN amplitudes for all stimuli [50]–[55], [57] or (2) show different error-likelihood effects (i.e. increased following EL and decreased following EF-learning) as postulated by the error-likelihood model for individuals with altered error learning rates [48], [49].

## Materials and Methods

The study was approved by the ethical committees of the Universities of Magdeburg and Lübeck.

### Participants

Sixteen German-speaking adults (six women, mean age 37.0) with the diagnosis of OCD as defined by DSM-IV criteria [61] and 16 neurologically and psychiatrically healthy control participants (six women, mean age 36.7) matched for age, school education and handedness participated after giving written informed consent. Table 1 gives the demographic and clinical characteristics of both groups.

**Table 1:** Detailed group characteristics of the obsessive-compulsive disorder group and the control group.

Patient no.	Sex	Age (years)	School (years)	H	OCS	Duration (years)	Y-bocs	BDI
1	m (m)	20 (18)	10 (10)	r (r)	Thinking	2	26 (14+12)	16 ( 7)
2	m (m)	23 (27)	10 (10)	l (r)	Symmetry/Ritual	2	26 (12+14)	10 (14)
3	m (m)	26 (25)	12 (12)	r (r)	Washing/Cleaning/Thinking	8	36 (18+18)	30 ( 3)
4	m (m)	28 (27)	10 (10)	r (r)	Washing	8	19 (9+10)	18 (27)
5	m (m)	29 (30)	12 (12)	r (r)	Thinking	2	32 (16+16)	24 ( 6)
6	m (m)	34 (34)	10 (10)	r (r)	Washing/Cleaning/Thinking	13	26 (11+15)	17 ( 0)
7	m (m)	42 (40)	10 (10)	l (l)	Cleaning/Thinking	20	28 (14+14)	18 (31)
8	m (m)	46 (45)	10 (10)	r (r)	Thinking/Symmetry	10	28 (13+15)	24 (10)
9	m (m)	50 (51)	10 (10)	r (r)	Thinking	7	34 (18+16)	25 ( 5)
10	m (m)	56 (58)	10 (10)	r (r)	Thinking/Writing	5	25 (15+10)	17 ( 4)
11	w (w)	26 (26)	10 (10)	r (r)	Thinking/Washing	2	25 (12+13)	12 ( 0)
12	w (w)	33 (32)	12 (12)	r (r)	Washing	10	33 (15+18)	18 ( 2)
13	w (w)	36 (34)	10 (10)	r (r)	Checking	9	34 (17+17)	19 ( 3)
14	w (w)	36 (38)	10 (10)	r (r)	Cleaning/Thinking	7	31 (17+14)	30 (14)
15	w (w)	52 (48)	10 (10)	r (r)	Thinking/Washing	25	34 (17+17)	24 ( 2)
16	w (w)	55 (54)	10 (10)	r (r)	Thinking/Writing	6	34 (18+16)	7 ( 1)

Information for the control subjects is given in parentheses. W, woman; m, man; H, handedness; r, right-handed; l, left-handed; OCS, obsessive-compulsive symptom; Medication: 1x Anafranil; 3x Citalopram; 1x Clomipram, Concerta; 1x Ergenyl chrono, Neurocil, Paroxetin, Zyprexa; 2x Fluoxetin; 4x Paroxetin; 1x Remergil; 1x Remergil, Seroquel, Tavor, Venlafaxin; 1x Stangyl; 1x Stangyl; Sertralin, Diazepam.

### Experimental procedure

Subjects participated in one EF-learning and one EL-learning session. The order of learning sessions was counterbalanced across subjects. One session comprised 6 runs each composed of a learning phase and a subsequent recognition phase. Each participant performed a word-fragment-completion task for 20 word-fragments [29]. In the EF condition, the first three letters of a word were given by the experimenter and the subject was asked to guess words to complete this fragment. The following example instructions were given by the experimenter: “I am thinking of a word that begins with the letters B-R-U”. The participant could have been guessed “Bruder” (brother). After the first answer the participant was required to have another guess, for example “Brust” (chest). After guessing some words (usually around 2–3 words), the experimenter revealed which word was the target word to be remembered. If subjects failed to guess the intended target word, the experimenter introduced example words and the target word. For each of the presented word-fragments at least two German words exist with a high and comparable guessing probability (German stem completion study, data courtesy Richardson-Klavehn and Düzel, unpublished), e.g. BRU: ‘Bruder’ [brother], ‘Brust’ [chest] [29]. Both of these words were produced with 34% probability in the German stem completion study. This triplet could have been competed with other but lower probability candidates, e.g. “Brunnen” (well, 13%), “Brunst” (ardour, 6%) or “Brutal” (brutal, 3%) [see also 29]. However, these words were not used during the recognition phase. For each fragment one high probability word was used during the learning phase as a target word, while another high probability alternative was used as distracter during the recognition phase. In the EL-learning condition the first three letters of the word were introduced by the experimenter directly followed by the target word. The EL trial was introduced as in the following example: “I am thinking of a word that begins with A–N–Z. This word is ‘Anzeige’ (advertisement, 53%)”. The subject had to repeat the target word immediately without guessing any additional words. Next to “Anzeige”, “Anzahl” (number, 28%) could have been another high probability word. This word was presented in the recognition phase as a non-target word for half of the subjects. The other half of the subjects had “Anzahl” as target in the EL condition with “Anzeige” being used as non-target during recognition. An example of stimuli assignment to the learning mode sessions per list is given in Table 2 [see also 29].

**Table 2:** Example of stimuli assignment.

Condition	List A	List B	List C	List D
Errorless target	Bruder	Brust	Anzeige	Anzahl
Errorless non-target	Brust	Bruder	Anzahl	Anzeige
Errorless new words	Imker	Tonne	Hafer	Olive
Errorful target	Anzeige	Anzahl	Bruder	Brust
Errorful non-target	Anzahl	Anzeige	Brust	Bruder
Errorful new words	Tonne	Imker	Olive	Hafer

English Translations: Bruder (brother), Brust (chest), Imker (beekeeper), Anzeige (advertisement) Tonne (tun), Hafer (oat), Olive (olive).

During EF-learning the participants guessed several words to complete the word-fragment which resulted in deeper processing of words as compared to errorless word list learning. To ensure such a deeper processing of words in the EL condition as well, participants had to produce a sentence with the word.

During each recognition phase, 20 targets, 20 distracters and 20 additional new words were presented in a randomized order [see also 31]. The task was to indicate by button press (right index/middle finger), whether or not a given word was a target word. The participants did not receive feedback about the correctness of the actual response. The words were presented in white letters on a black background in the middle of a computer screen. Stimuli subtended 0.57° in height and between 1.7° and 4.9° in width. The stimulus duration was 300 ms with a stimulus-onset-asynchrony between 1800 and 2500 ms.

### EEG recording and analysis

Electroencephalography and electrooculography signals were registered with a digitization rate of 250 Hz and filtered with a bandpass of 0.01–30 Hz. Twenty-nine tin electrodes mounted in an elastic cap were positioned according to the 10/20 system (Fp1/2, F3/4, C3/4, P3/4, O1/O2, F7/8, T7/8, P7/8, Fc1/2, Cp1/2, Po3/Po4, Fc5/6, Cp5/6, Fz, Cz, Pz). Bio-signals were re-referenced offline to the mean activity of two electrodes placed on the right and left mastoid. Eye movements were recorded in order to allow for later offline rejection. All electrode impedances were kept below 5 kΩ. Using individualized amplitude criteria on the electrooculography, trials with eye movement artifacts were excluded from further analysis. Response-locked brain potentials were averaged for epochs of 900 ms length with 300 ms baseline. The combination of learning mode (EF/EL), stimulus type (target, non-target, new word) and response (correct/incorrect) resulted in 12 different trial types. Because the frequency of false alarms for new words and false alarms for the EL modus was too low, these categories had to be neglected in the analysis (Table 3 for remaining trial types). The statistical analysis was performed using repeated measures designs as specified in the Results section. The target component for the evaluation of the brain potentials was the ERN. To evaluate the ERN repeated measures ANOVAs were conducted including the between-subjects factor Group (OCD vs. Control) and within subjects factors Learning Mode (EF vs. EL-learning), Response-type (hit, miss, new correct rejection), Electrode site (Fz, Cz) as within subject factors. This overall analysis was followed by more detailed ANOVAs as specified within the Result section. In order to evaluate the effect of error-likelihood an additional response factor (YES vs. NO responses) was included if applicable. Response-locked brain potentials were filtered with a 1–8 Hz bandpass filter prior to analysis. Mean amplitudes were calculated in the time window 0–100 ms (baseline −300 to 0) after response and entered into analyses of variance. For all statistical effects involving two or more degrees of freedom in the numerator, the Greenhouse-Geisser epsilon procedure was used to correct for possible violations of the sphericity assumption. Additionally, tests involving electrode x condition interactions were carried out on normalized data using the vector normalization procedure [62], [63]. Planned comparisons were calculated testing for differences between hits, false alarms and correct rejection within each learning condition as well as for response category differences between learning conditions.

**Table 3:** Description of experimental categories.

EF-Hits	Correct recognition of target guesses
EF-Misses	Non-recognized target guesses
EF-FA	False alarm to non-target guesses
EF-CR	Correct rejection of target guesses
EF-new CR	Correct rejection of new non-target words
EL-Hits	Correct recognition of old words
EL-Misses	Non-recognized old words
EL-CR	Correct rejection of distracter words
EL-new CR	Correct rejection of new non-target words

## Results

### Performance measures

Signal detection measures revealed that memory accuracy was significantly better for EL compared to EF-learning in both groups (Table 4). A Group (OCD vs. control) by Learning Mode (EF vs. EL) ANOVA on the signal detection measure *d'* revealed a clear main effect of Learning Mode (F(1,30) = 379, p<.0001), whereas neither the Group effect (F(1,30) = 0.23, not significant (ns)) nor the interaction Group by Learning Mode reached significance. The same pattern emerged for the measures *beta* (Group: F(1,30) = 0.84, ns; Learning Mode: F(1,30) = 7.24, p<.05; Group x Learning Mode: F(1,30) = 2.43, ns) and *criterion* (Group: F(1,30) = 0.08, ns; Learning Mode: F(1,30) = 263, p<.001; Group x Learning Mode: F(1,30) = 2.84, ns). Thus, both groups showed better performance measures for the EL learning compared to EF learning and no clear differences could be found between groups.

**Table 4:** Overview of Performance measure (LM effects).

		Performance			RT		
	Group	EL	EF	t-values	EL	EF	t-values
**hit**	OCD	83.0 (13.1)	56.1 (17.1)	6.67**	1055 (195)	1152 (242)	−1.43
	control	85.2 (7.8)	56.8 (9.6)	7.74**	969 (134)	1060 (131)	−3.05*
**misses**	OCD	13.7 (11.7)	39.6 (14.7)	−7.06**	1092 (197)	1214 (169)	−2.69*
	control	13.9 (10.2)	38.3 (7.9)	−6.48**	1075 (204)	1063 (122)	0.28
**CR**	OCD	89.1 (7.4)	56.3 (16.6)	8.42**	1082 (180)	1265 (336)	−2.39*
	control	90.3 (3.8)	61.4 (14.7)	8.96**	985 (131)	1076 (149)	−3.34*
**FA**	OCD	7.1 (5.8)	39.0 (16.6)	−8.36**	1143 (235)	1239 (143)	−1.55
	control	7.4 (3.9)	33.3 (14.5)	−8.28**	1109 (231)	1135 (206)	−0.54
**CRnew**	OCD	94.8 (4.4)	93.6 (6.7)	0.68	984 (179)	984 (194)	0.01
	control	95.8 (5.4)	94.0 (5.6)	1.29	911 (135)	885 (145)	1.60
**d'**	OCD	2.79 (0.65)	0.46 (0.26)	13.14**			
	control	2.6 (0.5)	0.6 (0.5)	14.67**			
**Beta**	OCD	3.29 (4.51)	1.04 (0.16)	2.03			
	control	2.1 (1.4)	1.2 (0.3)	2.48*			
**Precision**	OCD	1.59 (0.45)	0.32 (0.49)	11.47**			
	control	1.5 (0.3)	0.5 (0.4)	11.27**			

All df 15. OCD: Obsessive-compulsive disorder; RT: reaction times in msec; CR: Correct Rejection; FA: False Alarm.

The new words did not appear in the learning phase before and thus, should not be influenced by learning mode. Indeed, no significant effects were obtained for reaction times for new word correct rejections (ANOVA: Group: F(1,30) = 2.34, ns; Learning Mode: F(1,30) = 0.64, ns; Group x Learning Mode: F(1,30) = 0.61, ns), indicating no influence of learning mode on new words and no differences between both groups in terms of reaction times. However, reaction times for hits showed a significant main effect for Learning Mode (F(1,30) = 6.38, p<.05) but no Group (F(1,30) = 2.85, ns) or interaction effect (F(1,30) = 0.01, ns). In the pair-wise comparisons (Table 4) the Learning Mode effect on reaction times was only significant for the control group with faster reactions to EL compared to EF stimuli but no statistical difference was found for the OCD patients. For reaction times to misses both main effects did not reach significance (Group: F(1,30) = 2.37, ns; Learning Mode: F(1,30) = 3.25, ns) but we found a significant interaction (Group x Learning Mode: F(1,30) = 4.71, p<.05). Direct comparisons revealed that OCD-patients were slower in EF compared to EL trials but reaction times of the control group were similar for EL and EF trials (see Table 4). No differences were found for reaction times to false alarms (Group: F(1,30) = 1.19, ns; Learning Mode: F(1,30) = 2.46, ns; Group x Learning Mode: F(1,30) = 0.79, ns). Finally, reaction times to correct rejection yielded a main effect of Group (F(1,30) = 4.94, p<.05) and Learning Mode (F(1,30) = 11.39, p<.01) but no significant interaction. Tracing these effects by pair-wise comparisons showed that both groups showed faster reaction times for EL trials but that the control group showed overall faster responses (Table 4). Thus, reaction times to neutral stimuli (i.e. new words) were similar for both learning modes and both groups. However, responses to EL stimuli were faster as compared to EF trials for false alarms in both groups. The OCD patients showed faster responses to EL misses as compared to EF misses and no differences for hits whereas the control groups showed the opposite pattern, i.e. similar response times for misses in EL and EF learning and faster responses for EL hits as compared to EF hits.

### Response-locked brain potentials

**Figure 1:**
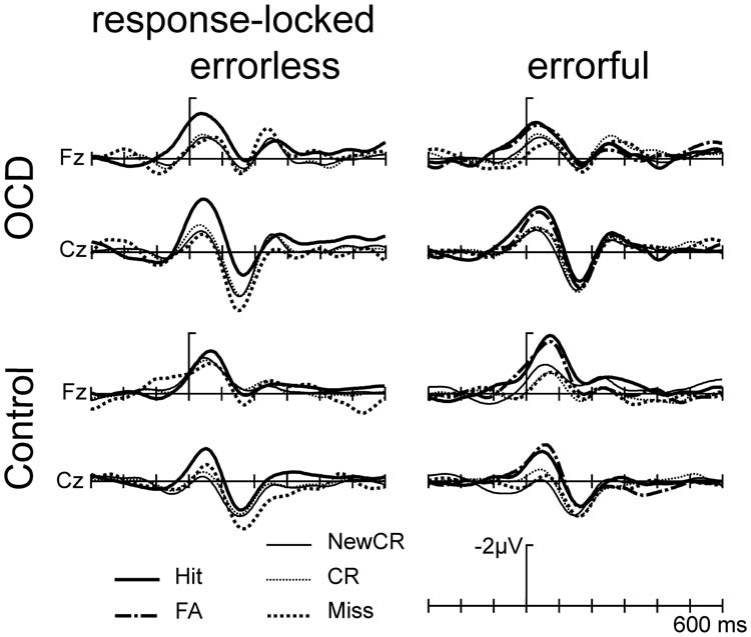
Response-locked potentials for OCD- and control-group. Response-locked ERPs (negativity is plotted up and each hash mark represents 100 ms of activity in this and in the following figures) of OCD patients (upper panel, N = 16) and control group (lower panel, N = 16). Hits related to both learning conditions and errorful false alarms result in an increased negativity compared to misses and both correct rejections. For the errorless mode (left panel) this is enhanced for OCD as compared to control group. For the errorful condition the opposite is true (most prominent at Fz).

**Figure 2:**
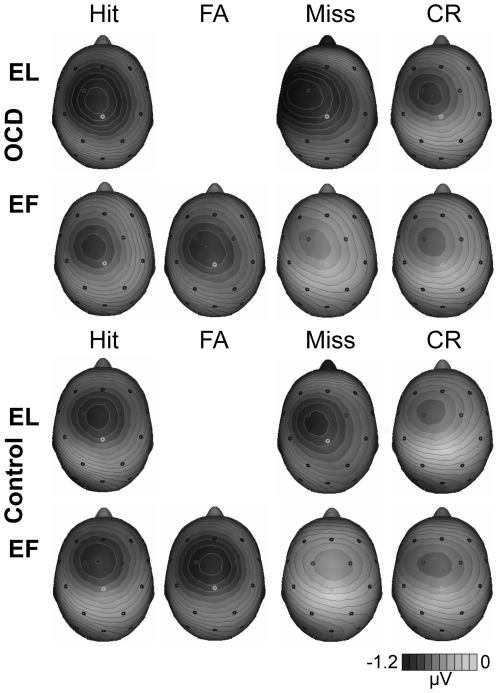
Topographical distributions of the brain potentials. Spline-interpolated isovoltage maps at 60 ms reveal a fronto-central distribution of the brain potentials. Darkest color is most negative.

**Figure 3:**
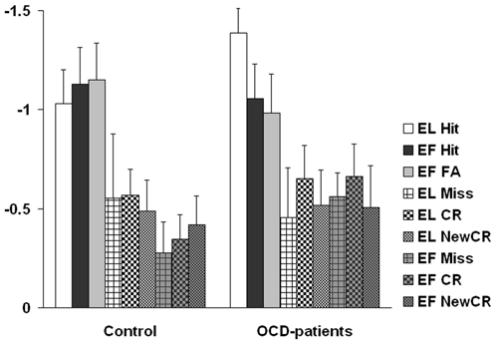
Mean amplitudes of the ERN. Bar graphs of mean ERP amplitudes at electrode sites Fz and Cz (0–100 ms after response) for the control groups (left) and OCD group (right).

Both groups showed a fronto-central negativity, which was most prominent for hits after EL and EF-learning as compared to new correct rejections and misses (Figure 1, for corresponding topographical maps see Figure 2 and for mean amplitudes see Figure 3). The distribution of this component suggests that it is an instance of the ERN. The overall ANOVA revealed consistent main effects for Learning Mode (F(1,30) = 35.43, p<.001) and Response-type (F(1,30) = 4.94, p<.05) and a Learning Mode x Response-type interaction (F(1,30) = 31.41, p<.001). The remaining effects did not reach significance.

Subsequently, ANOVAs were computed separately for the two learning modes (Group x Response-type x electrode site). In OCD-patients, the EL hits resulted in the largest ERN as compared to the other responses. This difference was not as pronounced for the control group (Figure 1, left panel). These findings were corroborated in a significant main effect for Group (F(1,30) = 9.3, p<.01) and a Group x Response-type (F(2,60) = 14.66, p<.001) interaction (Response-type (F(2,60) = 2.86, ns.). For the EF learning, an opposite direction was observed between groups: Here the EF hits resulted in an increased ERN within both groups but were enlarged for the control group compared to the OCD group (Figure 1, right panel). Statistically this was confirmed by the analogous ANOVA for the EF condition, which revealed a significant main effect for Group (F(1,30) = 6.64, p<.05), Response-type (F(2,60) = 5.5, p<.01) as well as an interaction between these two factors (F(2,60) = 29.67, p<.001). These results show a clear differentiation between both groups for both learning modes.

**Figure 4:**
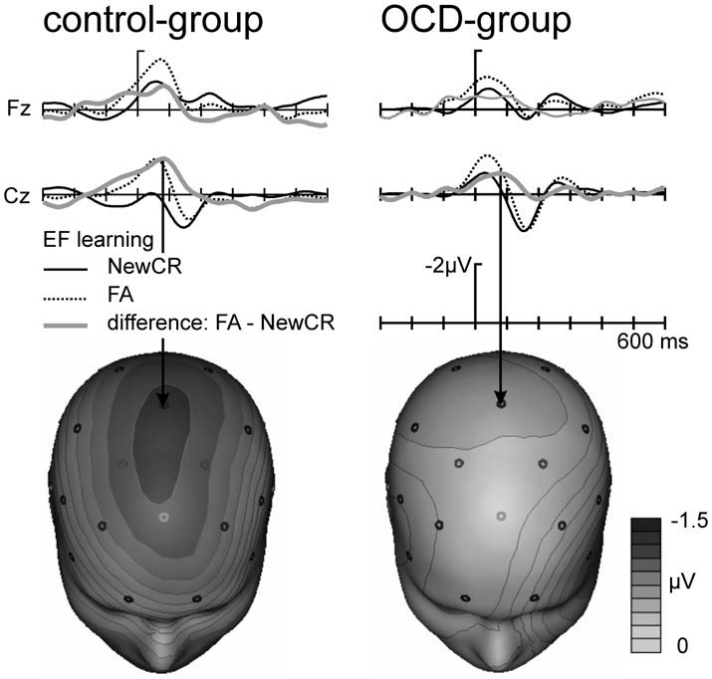
Difference waves of false alarms and correct rejections. Response-locked ERPs of false alarms in comparison to new correct rejection for the control (left panel) and OCD group (right panel). The grey line shows the difference wave of false alarm minus new correct rejections. The corresponding spline-interpolated isovoltage maps of the difference wave shows a fronto-central distribution for the control group. This effect is nearly absent for the OCD group.

EF-learning resulted in a sufficient number of false alarms. In both groups, EF false alarms were associated with an enlarged ERN response (right panel of Figure 1and Figure 4), which appeared to be smaller in the OCD group. An additional ANOVA including false alarms was performed with the factors Group and Response-type (2 levels: correct [hit, correct rejection] vs. erroneous [miss, false alarms]), Response (2 levels: yes [hit, false alarm] vs. no [miss, correct rejection]) and electrode site (Fz, Cz). Visual inspection suggested an increased negativity for yes responses compared to no responses in particular within the control group (see Figure 1 right panel, and Figure 3). Statistically, this was corroborated by a significant main effect for Response (F(1,30) = 91.37, p<.001) and a significant interaction between Group and Response (F(1,30) = 21.04, p<.001). All other effects were not significant (all df 1,30, all F<2.2). Planned pair-wise comparisons within groups (all df 1,15) were performed to trace back amplitude differences. Comparing the ERN amplitude of EL hits and misses we found a significant difference for the OCD group (F = 6.53, p<.05) but not for the control group (F = 1.93, p>.05). For EF-learning we observed the opposite: there was no significant difference for the OCD-group for EF hits vs. misses (F = 0.30, ns) and EF false alarm vs. correct rejection (F = 0.19, ns) but a significant difference for the control group (EF hit vs. misses: F = 5.28, p<.05, EF false alarm vs. correct rejection: F = 7.69, p<.05). Figure 4 illustrates this pattern: Whereas a clear differentiation between false alarm and new correct rejection was observed for the control group, this was absent for the OCD-group. Directly comparing the response types between both conditions (EL hit vs. EF hit, EL miss vs. EF miss, EL correct rejection vs. EF correct rejection, EL new correct rejection vs. EF new correct rejection) did not show significant differences (all F<1.45, p>.2).

## Discussion

OCD-patients and the healthy control group benefited from EL-learning as compared to EF-learning indicated by improved memory performance which is in accord with earlier studies showing enhanced memory performance for EL-learning [27], [29]. Contrary to our expectations, OCD patients as compared to the controls did neither show a decreased memory performance following EF-learning nor an enhanced memory performance following EL-learning. However, we found clear differential modulations of an early negativity obtained time-locked to the response in both groups. In line with previous electrophysiological investigations [29], [31], [64] we identified this negativity as an ERN based on its polarity, latency and topographical distribution (see Figures 1 and 2). There were significant differences between the two groups for the ERN to false alarm-trials in EF-learning and for hits from both, EL and EF conditions which hint at differences in the executive control of memory between OCD and control participants. In line with Heldmann et al. [31], we expected increased amplitudes for items with a high likelihood to commit an error following the postulations of the error-likelihood model [44], [46], [48], [49]. According to the error-likelihood model, it is not primarily the conflict or the error-detection that causes the activity in ACC observed as an ERN but rather the perceived probability of committing an error. The participants performed a word-list recognition task following EF- and EL-learning mode. In either case, the participant had to respond with YES if a word was recognized as a learned word and NO if it was recognized as a new word or distracter word. During the recognition phase there were twice as many non-target words (i.e. distracter words beginning with the same three letters and totally new words) than target words (i.e. learned words). In case of perfect recognition, the ratio of target words (requested yes-response) and non-target words (requested no response) was 1∶2 [see also 31]. The likelihood to commit an error with a YES-response (hit, false alarm) was twice as high as compared to a NO-response (correct rejection, miss). Heldmann and colleagues [31] found major differences of the ERN amplitude between yes- and no-responses. The error-likelihood model would predict such a difference: an increased ACC activity would be expected for all yes-responses compared to no-responses regardless of the correctness of response (Hit/false alarm) and the learning mode. Turning to the present data, this prediction from the error-likelihood model was borne out with yes responses from both learning modes associated with an increased negativity generally in both groups (see Figure 1).

However, there were marked differences between OCD patients and control subjects as illustrated by the bar graphs in Figure 3. The EL session resembles a standard wordlist recognition task. Here, the ERN to hits was enhanced in the OCD group compared to the control group. This result is in line with earlier reports of an enhanced ERN amplitude for OCD patients [50]–[57] and the hyperactivity of the ACC for OCD patients as shown by neuroimaging data [58], [59]. The amplitude enhancement has been interpreted as reflecting an overactive action monitoring system in OCD, an interpretation that is substantiated by an increased post-error slowing [55]. This interpretation also squares with the view that OCD is associated with a dysbalanced activity within cortical-striatal-thalamic-cortical circuits [65]–[69].

However, this effect was different following EF-learning which cannot simply be explained by the error-likelihood model. As outlined in the introduction, the guessed words during the learning phase might interfere with the learned words and produce a conflict in later recognition. The conflict monitoring theory [43], [70], [71] proposes that the ERN may reflect the degree of a response conflict between multiple response alternatives. Conflicting responses evoke a situation when errors are likely to be committed. Thus, following EF-learning there might be a “double impact” on ERN amplitude: an increased error-likelihood for yes responses and an increased conflict due to interfering false candidates after EF-learning. This leads to increased ERN amplitude for EF false alarms and hits compared to correct rejections for the control group (Figure 1 and 3). Intuitively one would expect a similar (if not even more pronounced) effect for OCD patients because of the frontal hyperactivity, specifically in ACC. However, for OCD-patients the opposite is the case. While OCD patients showed the largest ERN difference following the EL-modus, this difference is diminished after EF-learning (see Figure 4 for a direct comparison of EF false alarm and new correct rejection).

**Figure 5:**
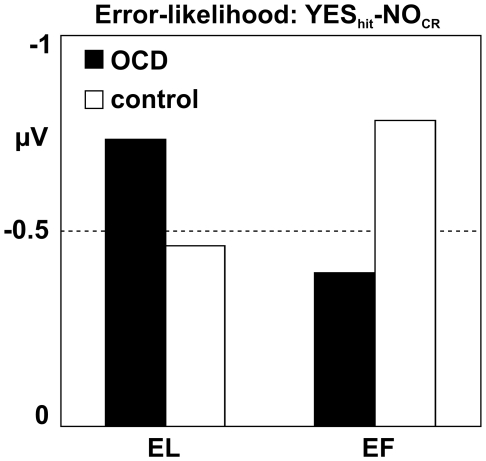
Error-likelihood effects. Error-likelihood effects of OCD and control participants. Bar graphs of the mean amplitude difference of correct Yes responses (hits) and No responses (correct rejections) at electrode sites Fz and Cz (0–100 ms) for EL-learning (reduced conflict) and EF-learning (high conflict).

Brown and Braver extended their model introducing individual differences based on different learning capabilities attributed to the ACC [48], [49]. In the following, we discuss our own results in the light of this extension. Here both, ‘YES’ and ‘NO’ responses resulted in a pattern as predicted by the error-likelihood model [44]. However, the influence of EF-learning had different impacts on both groups. Focusing on ‘YES’ responses, we observed increasing ERN amplitudes as the impact of conflict increases for the control group (lowest for EL hit and highest for EF false alarm). For the OCD-group the opposite picture emerged (highest for EL hit and lowest for EF false alarm, see Figure 1 and 4). This appears to be at odds with the notion of a hyperactive monitoring system in OCD patients which might lead us to expect increased ACC activity with a double impact of increased perceived likelihood and increased conflict. Brown and Braver's extended error-likelihood model [45], [49] is able to resolve these counterintuitive results: Individuals with altered ACC function (i.e. slow learning rate) showed reduced error-likelihood effects whereas response conflict was increased and vice versa for not-altered ACC functioning (i.e. fast learning rate). Thus, fast learning rates increase the efficiency of learning from errors, which increases the ability to predict an error at the expense of response conflict. The response conflict in our study consisted of two related effects: increased error-likelihood and increased number of possible responses. For the given design, the error-likelihood was constant over EL- and EF-learning as the ratio of YES- and NO-responses was the same for both learning modes. However, the amount of possible responses (here the amount of activated words depending on learning mode) differed: EF-learning introduced two alternative incorrect candidates which intervene in later recognition increasing response conflict compared to EL-learning (please note, however, that during recognition there might be still reduced conflict compared to EF based on incorrect memory traces). Figure 5 shows the error-likelihood effects for both groups (correct ‘YES’ responses (hits) minus correct ‘NO’ responses (correct rejection)) depending on the learning mode. None of the groups showed an absent or diminished error-likelihood effect as predicted by the model, which might be due to an increased cognitive load of the word list experiment as compared to the stop-and-change paradigm used by Brown and Braver (i.e. EL-learning is not purely conflict free but significantly less conflicting compared to EF).

However, the model is a good candidate to explain the effects for the EF modus. Following EF-learning, the OCD group showed a reduced error-likelihood effect as compared to the control-group (see Figure 5, right panel). This result was predicted by the extended error-likelihood model of Brown and Braver [48], [49] for altered ACC-functioning: slow ACC learning rates resulted in a decreased error-likelihood effect. Within a low conflict situation (recognition after EL-learning) OCD-patients compared to the control group show a considerable increase of the error-likelihood effect. This might be conceptualized as a hyperactive monitoring system [50]–[55], [57]. For high conflict situations (recognition after EF-learning) the opposite effect was observed: whereas the control group showed an increased error-likelihood effect, the OCD group showed a considerable reduction of the error-likelihood effect based on altered ACC learning rates in response to errors [48], [49]. This interpretation is supported by reports that OCD-patients compared to controls showed increased decision difficulties for simple or less risky situation (e.g. “seeing a piece of string on the ground”), whereas no differences were found for difficult or high risky decisions (e.g. “seeing a sharp wire in the parking lot”) [26] and might explain why OCD patients show decision difficulties in daily life for simple situations (‘Indecisiveness’ e.g. which detergent should be bought).

In conclusion, EL-learning enhances memory performance compared to trial-and-error learning (EF modus) in both groups. Differential ERN patterns of OCD patients and the healthy control group support the view of an altered conflict monitoring system and perceived error-likelihood effects in OCD.
